# Microbe-mediated nanoparticle intervention for the management of plant diseases

**DOI:** 10.1007/s44297-023-00006-9

**Published:** 2023-08-10

**Authors:** Temoor Ahmed, Jinyan Luo, Muhammad Noman, Munazza Ijaz, Xiao Wang, Hafiza Ayesha Masood, Natasha Manzoor, Yanli Wang, Bin Li

**Affiliations:** 1 State Key Laboratory of Rice Biology and Breeding, Ministry of Agriculture Key Laboratory of Molecular Biology of Crop Pathogens and Insects, Institute of Biotechnology, Zhejiang University, Hangzhou, 310058 China; 2 Xianghu Laboratory, Hangzhou, 311231 China; 3 Department of Plant Quarantine, Shanghai Extension and Service Center of Agriculture Technology, Shanghai, 201103 China; 4 Ningbo Jiangbei District Agricultural Technology Extension Service Station, Ningbo, 315033 China; 5 Department of Plant Breeding and Genetics, University of Agriculture, Faisalabad, 38000 Pakistan; 6 Department of Soil and Water Sciences, China Agricultural University, Beijing, 100193 China; 7 State Key Laboratory for Quality and Safety of Agro-Products, Institute of Plant Protection and Microbiology, Zhejiang Academy of Agricultural Sciences, Hangzhou, 310021 China

**Keywords:** Antimicrobial, Disease management, Microbial nanotechnology, Nanopesticides

## Abstract

Plant diseases are caused by various pathogenic microorganisms, leading to substantial economic losses and food insecurity worldwide. However, the extensive use of chemical-based nanopesticides has adverse effects on plants, soil, and environmental systems. There is increasing interest in developing eco-friendly and sustainable alternatives to manage plant diseases. Recently, microbe-mediated nanoparticles (NPs) as nanopesticides have attracted the interest of cultivators, specifically in plant disease management, compared to traditional physical and chemical approaches. This review focuses on the state-of-the-art formulations of nanopesticides by using microorganisms against bacterial and fungal phytopathogens. The article discusses the various mechanisms through which these microbes contribute to the enhanced effectiveness of NPs, including the production of bioactive compounds, improved nanoparticle synthesis, and the facilitation of targeted delivery. The review also highlights the advantages of using microbe-mediated nanopesticides, such as reduced environmental toxicity, increased biodegradability, and the potential to manage pesticide-resistant pathogens. Overall, the use of microbe-mediated NPs is an inexpensive, reliable, and eco-friendly approach for plant disease management.

## Introduction

The continuous increase in food scarcity due to the emergence of outrageous plant diseases has become a global issue [105]. In addition, escalating food demands are experiencing exponential growth as a direct consequence of the population explosion, exacerbating the prevailing circumstances. Consequently, it is imperative that we augment global food production by up to 70% by 2050 to effectively meet the nutritional requirements of the burgeoning global population [22]. Various phyto-pathogens, such as fungi, bacteria, and viruses, pose a significant threat to global food security by reducing crop productivity and causing substantial economic losses [122, 124]. Recently, the scientific community has been trying to devise efficient alternatives to remote techniques to expand food production with minimum impacts on the environment and agricultural soil properties [4]. Traditionally, chemical pesticides have been widely used to combat plant diseases, but their indiscriminate use has led to the emergence of more harmful pathogens, environmental pollution, and negative effects on nontarget organisms [43, 54]. Hence, there is an urgent need for eco-friendly approaches to increase agricultural productivity and manage plant diseases that could substitute the previously available orthodox techniques.

Recently, nanotechnology has obtained considerable recognition because of its substantial applications in the agriculture sector, particularly the use of nanoparticles (NPs) (size < 100 nm) for soil conditioning and plant disease management [37]. Additionally, NPs are inexpensive, effective substitutes for parent materials that have a high reaction rate, increased efficiency, and a high surface-to-volume ratio [44]. Several studies have reported that the use of NPs significantly increases plant growth and development by modulating nutrient availability and plant defense responses as well as by directly interacting with environmental stresses (either biotic or abiotic) under greenhouse and field conditions [11, 34, 82]. The production of NPs relies on chemical and physical processes, which are associated with several drawbacks, including high production rates, limited biocompatibility, substantial energy requirements, and the utilization of various toxic chemicals [71, 77, 95]. In the literature, several studies have revealed that the chemical synthesis of NPs has toxic impacts on plant, soil and environmental systems [40, 46, 66]. The application of chemically synthesized silver NPs exhibits phytotoxic effects by permeating plant tissues, inducing oxidative stress, and adversely affecting cellular activities, leading to reduced photosynthesis, altered nutrient uptake, and impaired root development [29, 64]. Furthermore, chemically manufactured NPs can also have ecological consequences, potentially affecting beneficial soil microorganisms and changing ecosystem dynamics [8]. These risks highlight the significance of cautious assessment and management when using chemically produced NPs in plant disease control techniques.

Conversely, microbe-mediated biosynthesis of NPs has great ability with regards to nontoxicity, easy scaling-up, long-term stability and eco-friendliness in comparison with NPs synthesized by chemical and physical methods [73]. Various microorganisms belonging to different categories (e.g., bacteria, fungi, yeast and microalgae) have been considered potential nanofactories for synthesizing metallic NPs [110]. The synthesis of microbe-mediated NPs involves using microorganisms and various techniques, such as green synthesis, bioreduction, and extracellular synthesis, to produce NPs with desirable properties [102]. These NPs have shown promising results in controlling a wide range of plant diseases caused by various pathogens, including *Fusarium oxysporum*, *Alternaria solani*, and *Xanthomonas oryzae* pv. *oryzae,* among others [2, 59, 80]. In the past, several studies have demonstrated that microbe-mediated NPs are less toxic to nontarget organisms and have lower environmental persistence than chemical pesticides [67, 100]. These benefits have prompted researchers to investigate the potential of microbe-mediated NPs as a sustainable and eco-friendly alternative for managing plant diseases.

The present review provides the current advancements in the microbe-based synthesis of NPs for plant disease control and covers the role of microorganisms in the biosynthesis of NPs and their potential uses in agriculture. Additionally, the article will discuss the interaction between microbe-mediated NPs and plant pathogens, highlighting the different modes of action.

## Microbe-mediated metallic nanoparticles

**Fig. 1:**
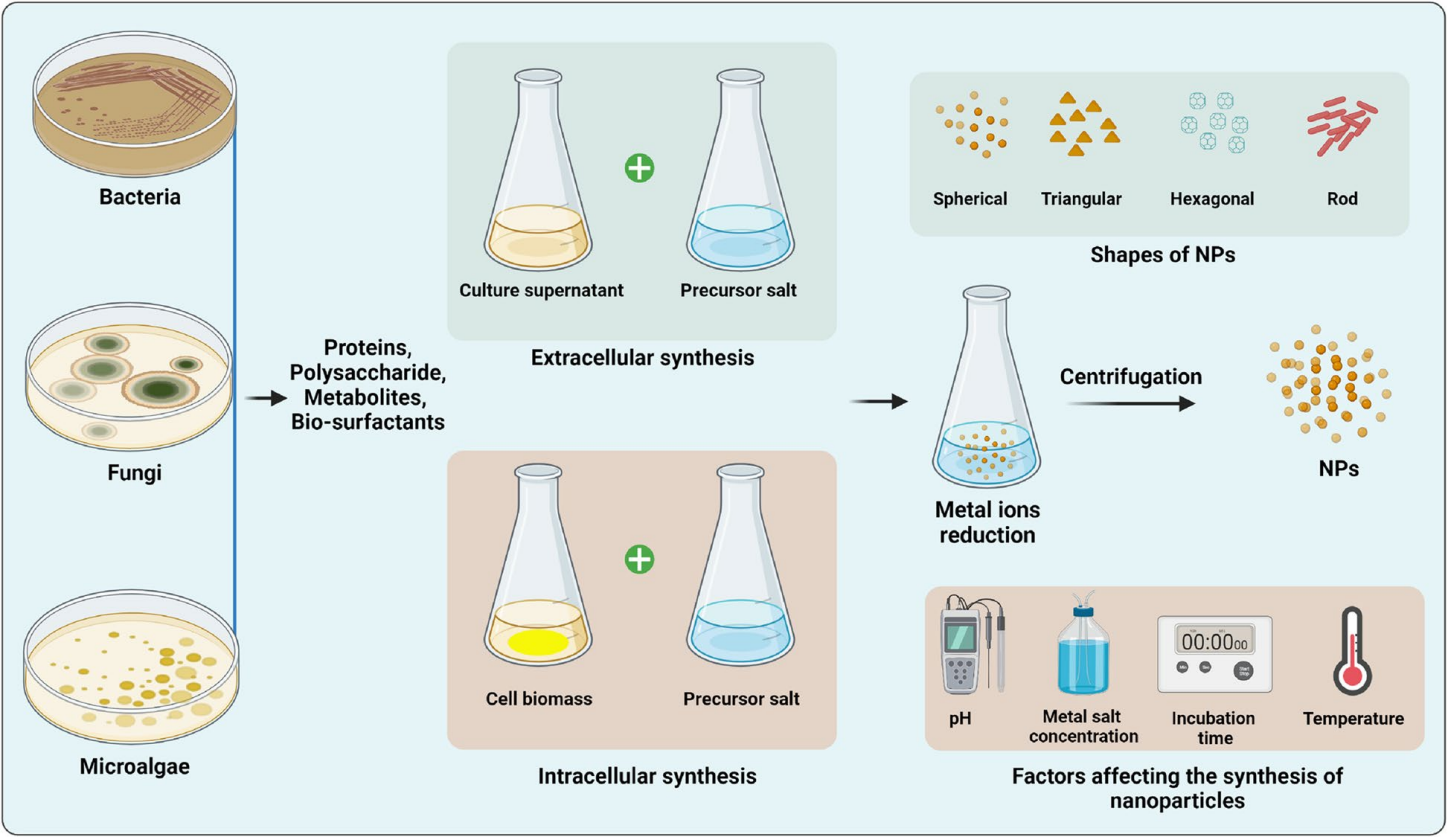
Schematic illustration of microbe-mediated synthesis of nanoparticles (NPs). Microbe-mediated synthesis of NPs involves the reduction and stabilization of metal ions by microorganisms such as bacteria, fungi, and algae. The process starts with the exposure of the microorganisms to metal ions, followed by the reduction of the ions to form NPs. The NPs are then stabilized by various biomolecules present in the microorganisms, such as enzymes and proteins. The shape of the NPs depends on various factors, including the type of microorganism, the reaction conditions, and the metal ions used

Nanoparticles have been manufactured using a variety of traditional chemical and physical techniques, such as the solvent evaporation process, vapor condensation, physical fragmentation, sol–gel process, precipitation from microemulsion, and interferometric lithography [5, 98]. These techniques involve using hazardous and toxic substances, which contribute to environmental pollutants. Furthermore, these toxic substances may bind with plants, causing NPs to accumulate in the food chain via food consumption, posing a risk to human health [60]. However, the microbe-mediated synthesis of NPs offers several benefits over traditionally used chemical processes, such as being eco-environmentally friendly, cost-effective, and biocompatible [107]. Microbe-mediated synthesis of NPs employing various microorganisms, such as bacteria, fungi, yeast, and microalgae, offers a promising approach due to its inherent ability to produce highly stable nanoparticles [18]. Furthermore, microbial synthesis allows for precise control over the size, shape, and composition of NPs, which can significantly impact their properties and potential applications [63]. The particle size, dispersion, and stability of biogenic NPs play crucial roles in determining their efficacy [69]. Numerous studies have highlighted the significance of these properties, showcasing the control and manipulation of particle size through microbial synthesis methods [106]. Additionally, the dispersion of microbially synthesized nanoparticles can be enhanced through surface modifications, allowing for improved interactions with target pathogens [92]. Furthermore, the stability of these nanoparticles, influenced by the capping agents produced by microorganisms, contributes to their sustained antimicrobial effect, as shown in Fig. 1.

### Synthesis of nanoparticles using bacteria

The production of NPs by using bacteria is a promising and eco-friendly approach to produce NPs with various applications in the agriculture sector [47]. Bacteria have been used to synthesize NPs because they can produce extracellular enzymes that have the ability to reduce metal ions to their corresponding NPs [51]. The synthesis of NPs using bacteria can be achieved through various mechanisms, such as intracellular biosynthesis, extracellular biosynthesis, and bioaccumulation [17, 121]. In intracellular biosynthesis, bacteria synthesize NPs within their cells. The process involves the uptake of metal ions into the bacterial cells and the reduction of the ions to NPs through the action of intracellular enzymes. The NPs are then released into the extracellular environment after cell lysis [31, 88]. Bacteria are used to synthesize NPs outside their cells in extracellular biosynthesis. The process involves the secretion of extracellular enzymes, which can reduce metal ions to their corresponding NPs. The NPs are then released into the extracellular environment, where they can be harvested and purified [112, 113]. In the literature, various studies have reported the green synthesis of metallic NPs using microbes [12, 86, 87]. Ahmed et al. [11] described the biosynthesis of silver NPs via *Bacillus cereus* to control the rice bacterial pathogen. Similarly, Varshney et al. [117] showed the green synthesis of copper NPs by using *Pseudomonas stutzeri*, which was isolated from wastewater. Several bacterial strains, viz., *B. amyloliquefaciens*, *Acinetobacter calcoaceticus*, *P. stutzeri, Escherichia coli* and *Lactobacillus* sp. were previously used for microbe-mediated synthesis of NPs [49, 65, 104, 111]. The green synthesis of copper, silver and zinc NPs from *Streptomyces* sp. has revealed that the reductase enzyme plays a vital role in reducing metal ions [62]. Taken together, the biosynthesis of NPs from bacteria is a promising and eco-environmentally friendly technique to produce NPs.

### Synthesis of nanoparticles using fungi

Fungi are a diverse group of organisms that have been widely used for the synthesis of NPs due to their ability to produce various extracellular enzymes and metabolites that can reduce metal ions to their corresponding NPs [38, 99, 119]. The biosynthesis of NPs from fungi can be attained through various mechanisms, including intracellular and extracellular biosynthesis and fungal biomass [78]. Intracellular biosynthesis involves the uptake of metal ions into fungal cells, which can be reduced to NPs through the action of different intracellular enzymes. The NPs are then released into the extracellular environment after cell lysis [101, 116]. Extracellular biosynthesis involves the secretion of extracellular enzymes that are able to reduce metallic ions into their corresponding NPs outside fungal cells [112, 123]. Many studies have reported that the fungal-based synthesis of NPs has several advantages, such as ease of cultivation, high production rate and low cost [96, 97]. For example, Tomah et al. [115] demonstrated the biosynthesis of silver NPs through the cell-free filtrate of *Trichoderma virens* HZA14 against the *Sclerotinia sclerotiorum* pathogen. Moreover, Singh et al. [109] showed that zinc oxide NPs were synthesized using *Aspergillus niger* against the *Alternaria solani* pathogen, which can cause early blight disease of potato (*Solanum tuberosum* L.) plants. Similarly, Jain et al. [56] reported the *Aspergillus aeneus*-mediated biosynthesis of zinc oxide NPs. Similar to other microbes, yeasts have also been generally applied for the biosynthesis of NPs on a large scale [19, 62, 79]. Taken together, the fungal-mediated synthesis of NPs offers a sustainable, cost-effective, and versatile approach for the green synthesis of NPs with unique properties and promising benefits in agricultural fields. Further research in this area could lead to the development of novel, eco-friendly technologies with a wide range of applications.

### Synthesis of nanoparticles using microalgae

The biosynthesis of NPs from microalgae has several benefits, such as ease of cultivation, high production rate and low cost [26]. In addition, microalgae can be easily harvested and processed to synthesize NPs. Moreover, using microalgae for nanoparticle synthesis is environmentally friendly, as it reduces the use of hazardous chemicals and reduces waste production [32]. Microalgae are photosynthetic microorganisms that can reduce metal ions to their corresponding NPs through the action of intracellular or extracellular enzymes [3]. Many studies have previously tested the synthesis of biogenic NPs using microalgae through intra- and extracellular biosynthesis methods [24, 72]. For example, da Silva Ferreira et al. [30] synthesized silver chloride NPs through the microalgal species *Chlorella vulgaris* and observed their antibacterial potential against pathogenic bacteria. Likewise, Çalışkan et al. [23] showed the synthesis of zinc, iron and silver NPs using the microalga *Galdieria* sp. and characterize the NPs through standard material characterization techniques, such as UV‒Vis spectroscopy and Fourier transform infrared spectroscopy (FTIR). Similarly, Salas-Herrera et al. [103] revealed the synthesis of copper NPs using the microalgae *Tetraselmis suecica*, *Dunaliella tertiolecta* and *Chlorella kessleri* under different conditions. In conclusion, synthesizing NPs using microalgae is a promising and eco-friendly approach for producing NPs. Further research is required for the optimization of the synthesis process and for understanding the underlying mechanisms of nanoparticle synthesis using microalgae.

## Mechanism of nanoparticles for controlling phytopathogenic diseases

Nanopesticides can be termed any plant protectant that contains nanomaterials as active ingredients to enhance disease control efficacy and biocidal properties [50, 61]. Nanoparticles have demonstrated remarkable efficacy as antibacterial agents against plant pathogens, owing to their exceptional microcidal activity. Introducing microbe-oriented NPs represents a novel approach in the prevention of plant pathogenic diseases. Numerous studies have shown that microbe-based NPs have great potential to replace traditional pesticides [28, 33, 68]. The microorganisms used in these NPs have numerous mechanisms of action, such as antibiosis, competition for nutrients and space, and induction of systemic resistance in plants. Additionally, the size, shape and surface characteristics of biogenic NPs can be engineered to target specific phytopathogens, making these NPs highly efficient and selective. The most widely studied metallic NPs are silver, gold, manganese, zinc, copper, titanium, etc. [74]. Overall, nanoparticle-mediated disease control mechanisms are complex, involving a variety of direct and indirect impacts on the pathogen, the plant, and their interactions. Further research is needed to fully understand the underlying mechanisms and optimize the efficacy and safety of nanoparticle-based approaches for the management of plant diseases. A summary of various types of microbe-based NPs against plant diseases is shown in Table 1.

**Table 1:** Summary of microbe-oriented NPs used as nanopesticides to manage plant diseases

**Microorganisms**	**Nanoparticles**	**Size (nm)**		**Shape**	**Applications**	**References**
**Bacteria**						
*Bacillus megaterium*	Mn	27–65	Spherical		Inhibited the Fusarium wilt disease in watermelon	[84]
*Bacillus altitudinis*	Cu	29–78	Spherical		In vitro and in vivo antibacterial activity against *Acidovorax citrulli *	[85]
*Bacillus aryabhattai*	FeO, BNCs	56, 86	Spherical		Control bacterial leaf blight disease by inhibiting *Xanthomonas oryzae* pv. *Oryzae* growth	[6]
*Paenibacillus polymyxa*	ZnO, MnO_2_, and MgO	62, 18, and 10	Spherical		Bactericidal effect against rice important pathogen *Xanthomonas oryzae* pv.* Oryzae *	[90]
*Acinetobacter johnsonii*	MgO	18–45	Spherical		Bactericidal activity against phytopathogen *Acidovorax oryzae *	[9]
*Enterobacter* sp.	ZrO	33–75	Spherical		Antifungal activity against bayberry pathogen *Pestalotiopsis versicolor *	[10]
*Aeromonas hydrophila*	ZnO	57–72	Crystalline		Antifungal activity against *Aspergillus flavus *	[57]
*Streptomyces griseus*	Cu	5–50	Spherical		Antifungal activity against tea pathogen *Poria hypolateritia *	[94]
*Streptomyces* spp.	CuO	78–80	Spherical		Antimicrobial activity against *Alternaria alternata, Fusarium oxysporum*, *Pythium ultimum* and *Aspergillus niger *	[41]
*Streptomyces capillispiralis*	Cu	4–59	Spherical		Antimicrobial activity against *Aspergillus niger*, *Fusarium* spp., Pythium spp. and *Alternaria* spp.	[42]
*Bacillus cereus*	Ag	18–39	Spherical		Bactericidal effect against rice important pathogen *Xanthomonas oryzae pv. oryzae *	[11]
*Pseudomonas rhodesiae*	Ag	20–100	Crystalline		Bactericidal activity against phytopathogen *Dickeya dadantii *	[45]
*Bacillus thuringensis*	Ag	10–20	Polymorphic		Antiviral activity against Sun hemp rosette virus	[55]
*Bacillus endophyticus*	Ag	4–26	Spherical		Antifungal activity against rice pathogen *Magnaporthe oryzae *	[52]
*Pseudomonas poae*	Ag	19–44	Spherical		Antifungal activity against Wheat pathogen *Fusarium Graminearum *	[53]
**Fungi**						
*Aspergillus oryzae*	Ag	40	Spherical		Antifungal activity against *Sclerotinia sclerotiorum *	[39]
*Trichoderma harzianum*	Ag	31.13	Spherical		Antifungal activity against *Sclerotium rolfsii* and *Sclerotinia sclerotiorum *	[35]
*Trichoderma virens*	Ag	5–50	Spherical		Antifungal activity against *Sclerotinia sclerotiorum *	[115]
*Fusarium solani*	Ag	5–30	Spherical		Antifungal activity against *Aspergillus* spp., *Fusarium* spp., *Rhizopus Stolonifera* and *Alternaria* spp.	[18]
*Guignardia mangiferae*	Ag	5–30	Spherical		Antifungal activity against *Rhizoctonia solani *	[20]
*Setosphaeria rostrata*	Ag	2–50	Spherical		Antifungal activity against *Rhizoctonia solani*, *Fusarium udum* and *Aspergillus niger *	[15]
*Aspergillus niger*	Ag	10–100	Spherical		Antifungal activity against *Fusarium oxysporum, Aspergillus Flavus* and *Penicillin digitatum *	[18]
*Fusarium oxysporum* f. sp.	Au	22	Spherical		Antimicrobial activity against phytopathogen *Pseudomonas* sp.	[114]
*Aspergillus terreus*	Zn	54–82	Spherical		Antifungal activity	[21]
*Trichoderma* spp.	ZnO	75	Spherical		Antibacterial activity against *Xanthomonas oryzae* pv. *oryzae *	[108]

### Direct interaction with pathogens

**Fig. 2:**
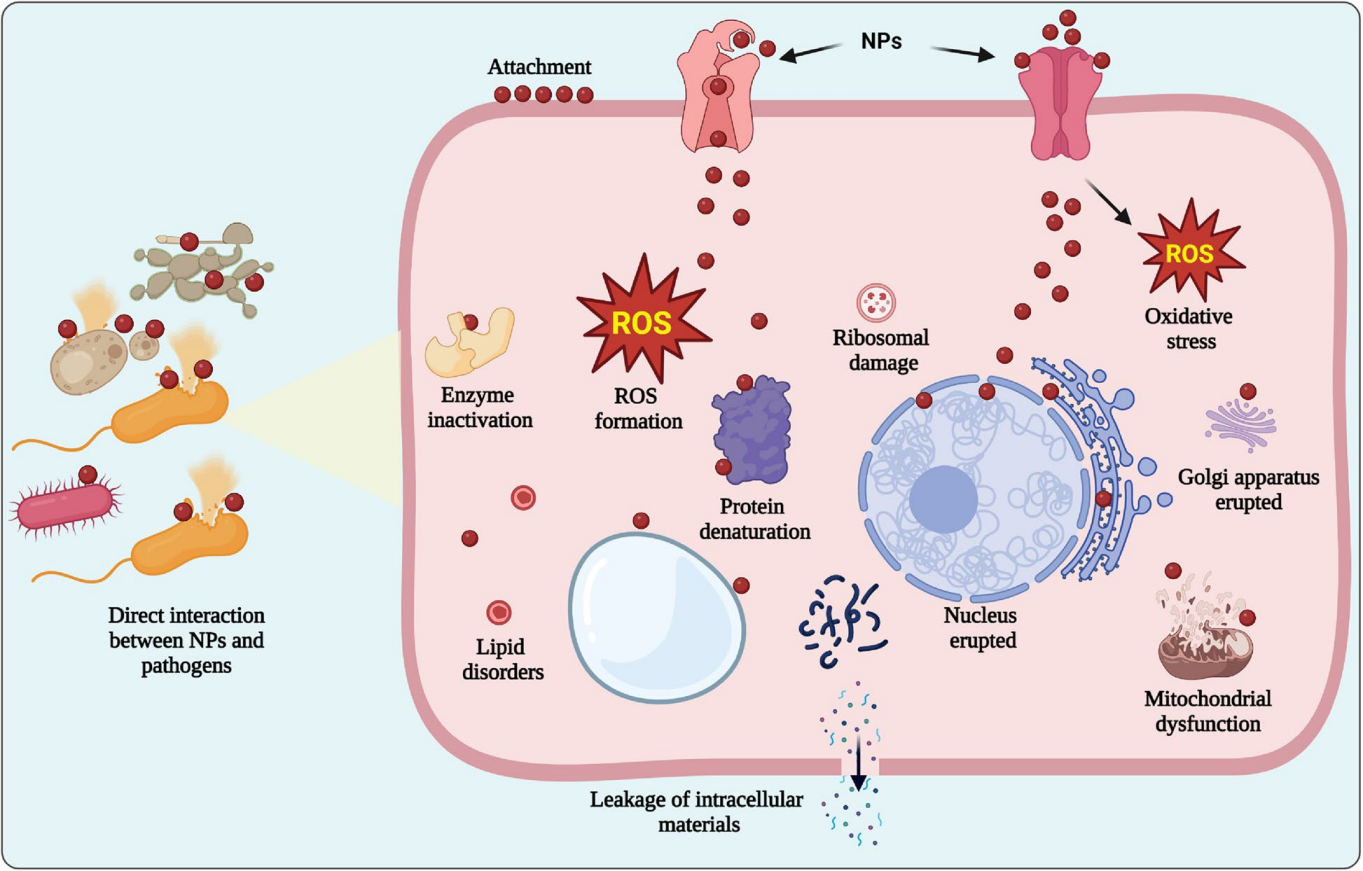
Schematic illustration of the direct interaction of nanoparticles (NPs) with plant pathogens. NPs can directly interact with phytopathogens, causing an oxidative burst that can lead to a cascade of events including cell wall damage, membrane disruption, denaturation of enzymes, and disruption of cellular functions, ultimately resulting in the death of the pathogen

Microbe-mediated NPs have shown promising results in directly interacting with plant pathogens, inhibiting their growth, and reducing disease severity (Fig. 2). One of the mechanisms by which biogenic NPs interact with plant pathogens is through their physical and chemical properties [83, 118]. These NPs may contain compounds that disrupt the pathogen’s cell membrane, leading to cell lysis and reduced pathogen viability. Additionally, the production of reactive oxygen species (ROS) (e.g., hydrogen peroxide, superoxide anions and hydroxyl radicals) after exposure to NPs causes DNA damage, inhibiting mRNA and protein synthesis that ultimately leads to pathogen death [89]. Several studies have suggested that a large surface area, nanosize scale, easy cell penetration and other distinct characteristics of biogenic NPs can significantly increase their antimicrobial activities [18]. The controlled release of protein-capped metal ions such as Cu^2+^, Ag^+^, Ti^4+^ and Zn^2+^. from nanocrystals are proposed in antimicrobial mechanisms [13, 16]. Hence, the interaction between NPs and microbial cells disrupts the cellular membrane structure, depletes antioxidants and interferes with nutrient uptake by microbes [7].

For example, Chen et al. [27] revealed strong antimicrobial activity produced by green MgO-NPs against *Thielaviopsis basicola* and *Phytophthora nicotianae*. They also observed that direct interactions between NPs and fungal cells triggered the production of ROS due to NP-associated damage to fungal cells. Furthermore, ultrastructural micrographs showed plasmalemma disappearance, partial cell wall injury and disorganized cytoplasm. Similarly, Ahmed et al. [9] demonstrated that green MgO-NPs have strong inhibitory effects on *A. oryzae* by using TEM analysis and showed a highly ruptured cell membrane structure, DNA damage and efflux of cytoplasmic materials that cause bacterial death. In a recent study, green-synthesized NPs were tested against the fungal pathogen *F. graminearum,* and the fungicidal effect causing alteration of hyphal and highly damaged cell wall structures was studied by electron microscopy analysis, e.g., SEM and TEM [52]. Similar effects of bioengineered chitosan-magnesium nanocomposites against the rice fungal pathogen *R. solani* and bacterial pathogen *A. oryzae* were also observed. Microscopic images showed extremely wounded structures of the cell membrane and cell wall of pathogens, cellular organelle damage and leakage of cytoplasmic materials after treatment with nanopesticides [7].

In a previous study, Hossain et al. [45] demonstrated the inhibitory ability of *P. rhodesiae*-mediated AgNPs to kill *Dickeya dadantii* (soft rot pathogen). In a recent study, Ibrahim et al. [51] found that green silver NPs synthesized using *B. siamensis* showed a proficient bactericidal effect against the rice pathogen *Xanthomonas oryzae* pv. *oryzae* that can cause bacterial leaf blight disease in rice. In another study, Kumari et al. [70] reported the in vitro fungicidal effect of biologically synthesized silver NPs against *Alternaria solani* by inhibiting spore germination and reducing biomass by 100% after 7 days. Similarly, another in vivo study revealed the inhibitory effect of three biologically synthesized metal oxide NPs (MgO, ZnO and MnO_2_) by *Paenibacillus polymyxa* against the rice bacterial leaf blight pathogen *Xanthomonas oryzae* pv. *oryzae* [90]. Recently, many studies have also determined the use of NPs for the control of viral diseases. For example, a study described the use of silver NPs synthesized from *Pseudomonas fluorescens* to control tobacco mosaic virus in tobacco plants [14]. However, extensive research is needed to enhance our understanding of the target specificity of biogenic NPs by assessing their effects on beneficial microorganisms.

### Activation of plant defense responses

**Fig. 3:**
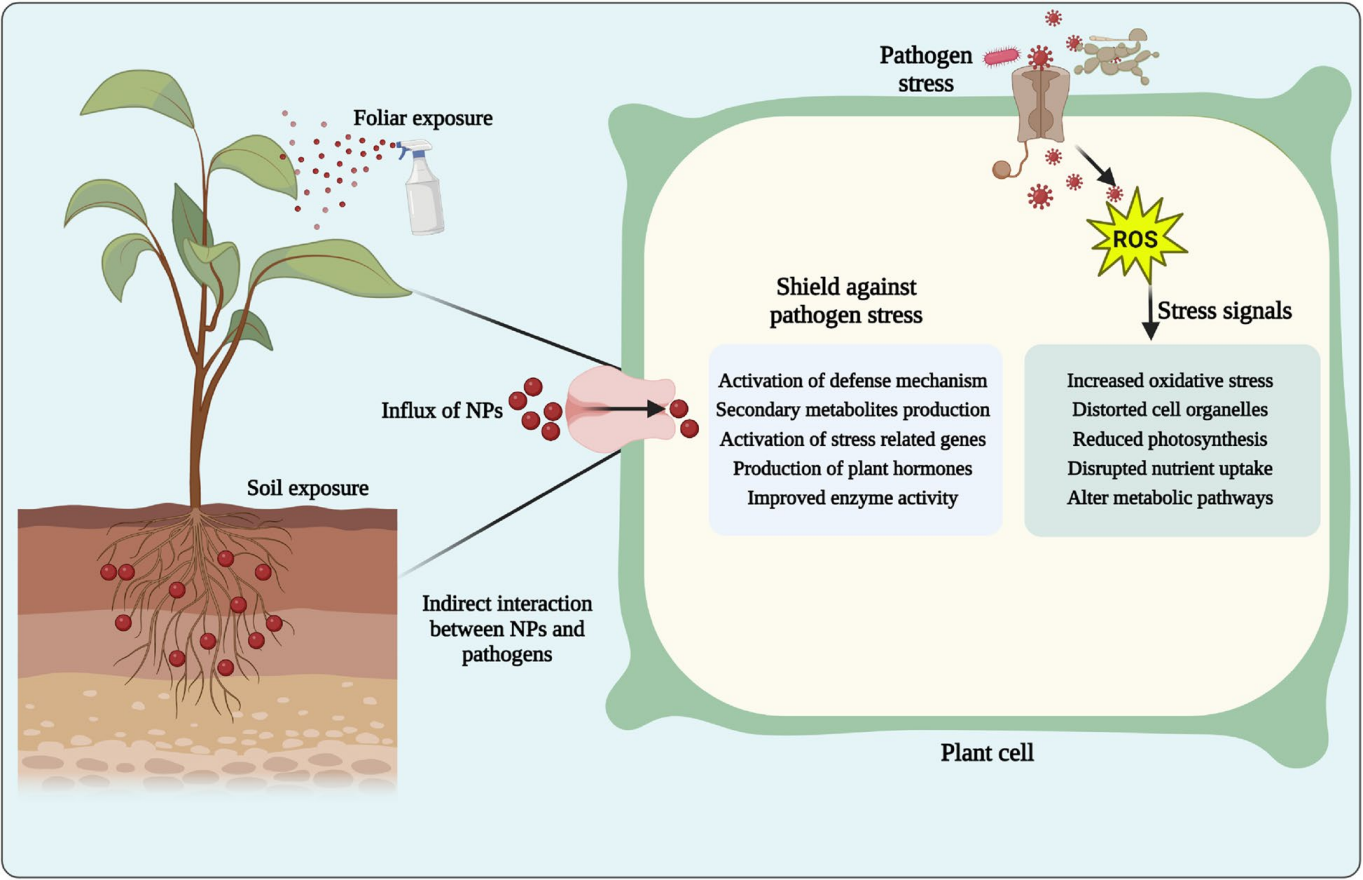
Schematic illustration of indirect interaction of nanoparticles (NPs) with phytopathogens. NPs can interact with the plant host and modulate the plant defense response, which can ultimately affect the pathogen’s ability to infect and cause disease. NPs can stimulate the production of plant defense molecules, including antioxidative enzymes, metabolites, phytohormones and pathogenesis-related proteins, and reduce oxidative stress, which can enhance plant resistance to pathogen infection

Microbe-mediated NPs have shown potential in indirectly controlling plant disease through their effects on plant growth and defense mechanisms (Fig. 3). For example, some NPs have been shown to increase the uptake and utilization of nutrients by plants, leading to improved growth and yield [25]. This can lead to the production of phytohormones, enzymes, and other defense molecules that inhibit pathogen growth and improve plant resistance to infection. Another mechanism by which biogenic NPs indirectly control plant disease is through their effects on plant defense mechanisms [58, 85]. Some NPs have been shown to induce the production of reactive oxygen species (ROS) in plants, which can trigger defense responses and reduce disease severity [120]. Additionally, NPs may stimulate the production of plant hormones such as salicylic acid and jasmonic acid, which play key roles in plant defense against pathogens [36, 75]. Previously, many studies have demonstrated the potential of biogenic NPs in controlling plant disease [81, 91].

For instance, biogenic copper NPs have demonstrated potential in suppressing bacterial fruit blotch in watermelon because they have direct antibacterial activity and can induce active immunity in watermelon [85]. Similarly, Cu nanoscale (250 mg L^−1^) amendments significantly suppressed soybean sudden death syndrome by activating plant immunity and enhancing the phytohormone content, photosynthetic endpoints, antioxidant enzymes and nutritional status [76]. In addition to their direct effects on plant growth and defense mechanisms, biogenic NPs can interact with soil and rhizosphere microorganisms to indirectly control plant disease [1, 93]. Furthermore, the use of chitosan-iron nanocomposites (BNCs) has shown promising results in controlling bacterial leaf blight disease in rice because it can inhibit *Xanthomonas oryzae* pv. *oryzae* growth and improve plant resistance through modulation of antioxidant enzymes, defense-related genes, and the plant’s microbiome [6]. Additionally, NPs may have antimicrobial properties that can inhibit the growth of pathogenic microbes in the soil, reducing the risk of disease [48]. Recently, Noman et al. [84] showed that biogenic manganese NPs (MnNPs) synthesized by *Bacillus megaterium* NOM14 have the potential to suppress watermelon Fusarium wilt through multiple mechanisms, including inhibition of pathogen growth, enhancement of the host defense response, and modulation of the soil microbial community. Overall, biogenic NPs offer a promising approach to the indirect control of plant disease by enhancing plant growth and defense mechanisms. However, further research is needed to fully understand their safety and environmental impacts and to optimize their use in agriculture.

## Concluding remarks and future perspectives

In conclusion, the use of nanotechnology in agriculture, specifically in the development of NPs, is a promising approach to manage plant diseases. Microbe-mediated NPs have emerged as an innovative and effective approach to plant disease management due to their potential for targeted delivery and enhanced efficacy. The use of microorganisms in the production of NPs has the potential to address some of the limitations of traditional chemical-based pesticides, such as their nonspecificity and harmful effects on the environment. Microbial-based NPs can target specific pathogens and reduce the amount of pesticide needed, thereby minimizing environmental contamination. However, there are still significant limitations and knowledge gaps to be filled to guarantee the social acceptance of NPs under environmental conditions as well as their safe use. Optimization of synthesis, ensuring NP stability and bioavailability, and achieving efficient delivery to target sites are key challenges. Additionally, concerns regarding the ecological impact and safety of NPs need to be addressed.

Pioneering efforts are needed to optimize biological synthesis methods on an industrial scale with benefits, including an eco-friendly nature, ease of scaling up and cost-effectiveness. Although NPs have demonstrated potential applications in agriculture, new tools for smart delivery of nanopesticides should be designed and commercialized. One of the potential areas of research is the integration of these pesticides with other innovative technologies, such as precision agriculture and gene editing. Precision agriculture can help farmers optimize the use of these pesticides by providing real-time information on plant health and disease prevalence. One of the primary challenges is the lack of knowledge and awareness among farmers and researchers about the use and effectiveness of these pesticides. Additionally, the regulatory framework for these pesticides needs to be developed to ensure their safety and efficacy. Moreover, field experiments are required to govern the effectiveness, steadfastness, reproducibility, and fate of microbe-mediated NP effects under realistic agricultural conditions. Notably, it is important to confirm that nanoscale pesticides do not adversely impact the growth of plants, beneficial microbial communities, or environmental processes. Further research is needed to address these challenges and fully realize the potential of microbe-mediated NPs. We believe that our review would be a constructive addition to sustainable agricultural systems to develop novel NPs for effective and low-cost management of plant pathogens to achieve global food security.

## Data Availability

All data used in this study are included in this article.
